# Efficient Tissue Clearing and Multi-Organ Volumetric Imaging Enable Quantitative Visualization of Sparse Immune Cell Populations During Inflammation

**DOI:** 10.3389/fimmu.2020.599495

**Published:** 2021-01-25

**Authors:** Julian Hofmann, Iana Gadjalova, Ritu Mishra, Jürgen Ruland, Selina J. Keppler

**Affiliations:** ^1^Institute for Clinical Chemistry and Pathobiochemistry, München rechts der Isar (MRI), Technical University Munich, Munich, Germany; ^2^TranslaTUM, Center for Translational Cancer Research, Technische Universität München, München, Germany

**Keywords:** 3D imaging, ethyl 3-phenyl-2-propenoate (ECI), inflammation, glomerulonephritis, histo-cytometry

## Abstract

Spatial information of cells in their tissue microenvironment is necessary to understand the complexity of pathophysiological processes. Volumetric imaging of cleared organs provides this information; however, current protocols are often elaborate, expensive, and organ specific. We developed a simplified, cost-effective, non-hazardous approach for **e**fficient tissue clearing and **m**ulti-**o**rgan **v**olumetric **i**maging (**EMOVI**). EMOVI enabled multiplexed antibody-based immunolabeling, provided adequate tissue transparency, maintained cellular morphology and preserved fluorochromes. Exemplarily, EMOVI allowed the detection and quantification of scarce cell populations during pneumonitis. EMOVI also permitted histo-cytometric analysis of MHC-II expressing cells, revealing distinct populations surrounding or infiltrating glomeruli of nephritic kidneys. Using EMOVI, we found widefield microscopy with real-time computational clearing as a valuable option for rapid image acquisition and detection of rare cellular events in cleared organs. EMOVI has the potential to make tissue clearing and volumetric imaging of immune cells applicable for a broad audience by facilitating flexibility in organ, fluorochrome and microscopy usage.

## Introduction

During immune homeostasis and inflammatory challenges, the communication between cells is crucial for a concerted immune response. This communication relies to a large part on the precise positioning of individual cell types in specific anatomical locations (the immune niche) (1, 2). It is thus essential to gain spatial information of cells as well as their distribution within their respective tissue niches in order to understand and manipulate cell-cell and cell-niche communication. Nevertheless, our current understanding of these processes still mainly relies on single-cell analysis of cells that are easily extracted from dissociated or lysed tissue. In this process, information such as cellular interactions, behavior, and localization is lost.

The accurate study of cellular organization in tissues requires a method permitting the visualization of larger tissue volumes revealing three-dimensional (3D) associations of cells and their surroundings. However, conventional imaging techniques, such as immunofluorescence or histology, usually analyze only a small part of an organ often in 2D, are limited by the selected sections and suffer from involuntary field of view selection bias. In addition, many cell types are rare and difficult to detect within these individual sections. Volumetric imaging of cleared large tissue pieces or whole organs is a valuable alternative to traditional immunofluorescence or histology of thin tissue sections. A multitude of optical clearing methods, such as BABB, CLARITY, CUBIC, iDISCO, Ce3D, BALANCE, and several improved versions of these have been developed for this purpose (3–14). All of these techniques try to reduce absorption and scattering of light through the tissue by homogenizing the refractive index (RI) of a sample [reviewed in (15, 16)]. In addition to being time-consuming, the main disadvantage for most of these applications is the use of toxic and/or corrosive chemicals that might be harmful to the researcher or damage the microscope. To detect immune cells in a variety of tissues in 3D, most immunological studies use transgenic mice expressing fluorescent reporters (14, 17, 18), or intra-venous (i.v.) injections of antibodies into living mice (13, 19). Although this is the fastest way to obtain a homogenous staining, disadvantages are the need for an animal license, high concentrations of antibodies, limited multiplexing, and the prevention of using several organs of the same animal for different stainings.

In order to overcome these limitations, we combined several recently published protocols (13, 14, 19, 20) and improved the methodology to develop **EMOVI** (**e**fficient tissue clearing and **m**ulti-**o**rgan **v**olumetric **i**maging). EMOVI is a simplified, cost-effective and non-hazardous whole-mount imaging approach that can be applied to a multitude of organs. Our results demonstrate that EMOVI enables multiplexed antibody-based immunolabeling, provides adequate tissue transparency, and maintains cellular morphology. We found that EMOVI preserved fluorochromes over several months, which adds to the flexibility in microscopy usage. We further demonstrate that EMOVI can be used to image and quantify changes in sparse cell populations in the lung during systemic inflammation. Furthermore, we found that EMOVI permits volumetric multiplex imaging and quantitative histo-cytometry as measure of disease activity in nephritic kidneys. We used EMOVI to compare light sheet fluorescence microscopy, widefield and confocal microscopy and found that widefield microscopy with real-time computational clearing might be a valuable alternative to confocal microscopy for rapid image acquisition. Thus, our EMOVI approach will make the detection of immune cells in cleared tissue applicable for most organs with equipment (reagents for staining, confocal microscope) commonly available or accessible in an immunological research environment.

## Results

### Workflow and Timeline of Our EMOVI Approach

**Figure 1:**
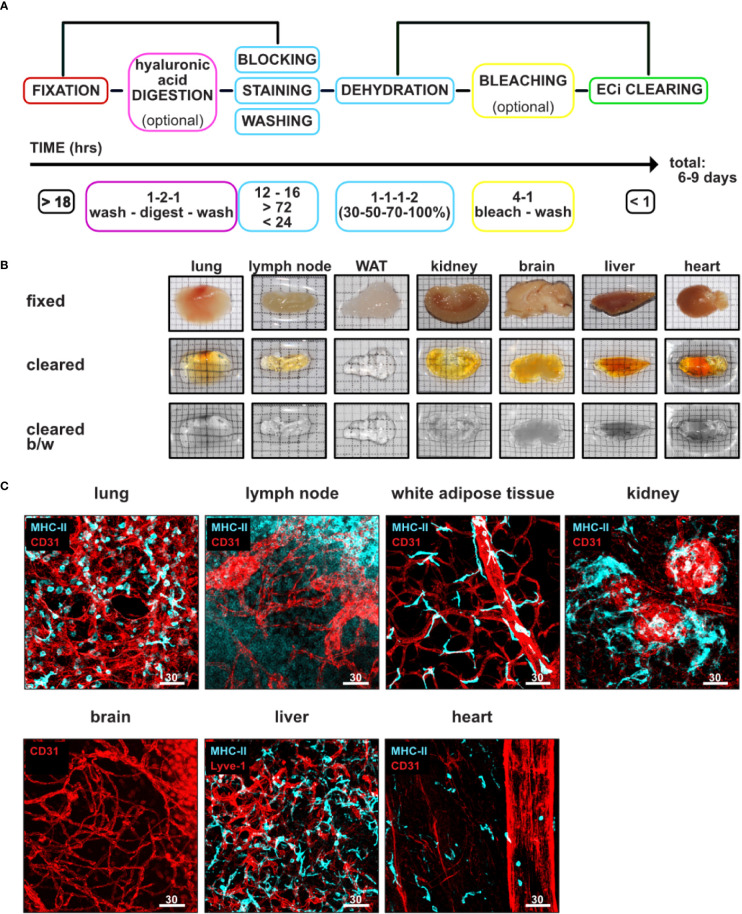
Workflow and timeline of our EMOVI approach. **(A)** Workflow and timeline describing our “one method fits all” approach for multiple organs. **(B)** Images of indicated fixed tissues taken before and after ECi clearing. Images of cleared organs depicted in color and black and white (b/w) images to better display transparency. WAT = white adipose tissue. **(C)** Images of fixed tissues stained with the indicated antibodies, cleared and z-stacks imaged by confocal microscopy. Scale bars in µm. Identical images of the kidney and heart have been used in **Figure S1C**. Representative images of at least three independent experiments are shown.

In order to achieve multiplexed antibody-based immunolabeling with good tissue transparency and cellular morphology, EMOVI combines several recently published protocols (13, 14, 19, 20) (**Figure 1A**, for detailed information please refer to our *Material and Methods* section). In order to reduce unspecific labeling and improve tissue penetration of the antibodies, EMOVI uses a saponin-containing fixative (IC Fixation buffer) for all organs not only the brain as previously described (14). Furthermore, we implemented the digestion of parts of the extracellular matrix (ECM) by hyaluronidase for more challenging organs with high ECM density, such as the kidney, liver, brain and heart (as indicated in **Table 1**). Hyaluronidase removes hyaluronic acid—a glycosaminoglycan which structures tissue architecture and increases viscosity and hydration in interstitial collagenous matrices. Hyaluronidase treatment improved signal intensity as well as penetration depth of the antibodies (**Figures S1A, B**. Glomeruli area and structure were similar in H&E stained sections of hyaluronidase treated or control kidneys, indicating that structural components of the organs were not affected (**Figure S1C**). To obtain evenly distributed immunolabeling, we here only used fluorochromes with high stability and good tissue penetrance (Alexa Fluor dyes) directly coupled to primary antibodies, as suggested by recently published protocols (4, 21). For blocking, staining, and washing of the organs, we used buffers with a mild detergent. Washing the samples with a buffer containing thioglycerol improved decolorization by removing endogenous pigments, such as heme (7, 14, 21). Dehydration is a critical step before tissue clearing and RI matching. We found that dehydration of the samples with pH-adjusted isopropanol preserved all fluorochromes tested better than ethanol in accordance of published data using 1-propanol (20). Dehydration with isopropanol resulted in tissue shrinkage from approximately 20% to approximately 60% of the total volume, dependent on the tissue (**Table 1**). After dehydration, we added an additional bleaching step using peroxide (13). Equilibrating tissue autofluorescence by using peroxide improved tissue penetration of the excitation laser. This bleaching step was previously used to maximize the signal quality of i.v.-mediated staining, but quenched the endogenously expressed fluorescent reporter dtTomato (13). The authors did not test peroxide on tissues immunolabeled with fluorescently-coupled antibodies after fixation of the organs. In our hands, freshly prepared peroxide reduced the fluorescence intensity slightly when staining abundant epitopes such as CD31 in the kidneys (**Figures S1A, B**). However, we sometimes experienced bleaching in other tissues immunolabeled after fixation (such as lung), which was prevented when using solutions that were prepared a week or longer before tissue treatment.

**Table 1:** Table specifying size, treatment, shrinkage by dehydration and approximate clearing time of tissues shown in **Figure 1**.

organ:	lung	lymph node	fat	kidney	brain	liver	heart
**tissue**	**1 lobe**	**complete**	**thin piece**	**half a kidney or 1 mm slice**	**1 mm slice**	**thin piece**	**half**
**hyaluronic acid digestion**	**NO**	**NO**	**NO**	**YES**	**YES**	**YES**	**YES**
**bleaching**	**NO**	**NO**	**NO**	**YES**	**YES**	**YES**	**YES**
**shrinkage**	**ca. 27%**	**ca. 34%**	**ca. 35%**	**ca. 44%**	**ca. 55%**	**ca. 41%**	**ca. 20%**
**clearing time**	**<30 min**	**10 min**	**<30 min**	**<60 min**	**<60 min**	**<60 min**	**<60 min**

We decided to use the organic compound ethyl cinnamate (Ethyl 3-phenyl-2-propenoate; ECi) for RI matching and tissue clearing due to its non-toxic nature, inexpensive availability, and flexibility in organ usage (13, 17, 19, 20, 22). We found that ECi cleared every tissue analyzed within 1 h (**Table 1**). Despite bleaching, ECi cleared tissues still demonstrated a brownish color (**Figure 1B**); however, this did not influence the penetration depth of the laser further. From harvesting of the organs to image acquisition, our here described improved protocol for multiplex antibody labeling and tissue clearing required about a week for organs such as the lung, lymph nodes or white adipose tissue (WAT) and a maximum of 9 days for more challenging organs such as the kidney, brain, liver and heart (**Figure 1A** and **Table 1**).

We next compared EMOVI immunolabeling and tissue clearing of kidneys to BALANCE (13) and Ce3D (21). BALANCE is an ECi-based technique that enables imaging of cells expressing fluorescent reporters or structures labeled by i.v. injection but has not been tested for clearing organs immunolabeled post-fixation. Ce3D has been established to label immune cells in several organs post-fixation followed by clearing using a mix of chemicals. Changes of EMOVI to BALANCE and Ce3D protocols are compared in **Table 2**. EMOVI-treated samples displayed higher fluorescence intensity and better signal-to-background ratio compared to BALANCE or Ce3D protocols, as demonstrated by the CD31-stained glomeruli throughout half a kidney using widefield microscopy (**Figure S2A**). Using confocal microscopy, we found that samples stained and cleared according to our EMOVI approach demonstrated an improved penetration depth of the antibodies especially compared to Ce3D (**Figure S2B**) as well as a better signal-to-background ratio and enhanced display of the glomeruli structure compared to the BALANCE protocol (as quantified by fluorescent intensity in areas of glomeruli and background (**Figure S2C**). The advantages of EMOVI compared to BALANCE and Ce3D hence are the improved staining of organs post-fixation, a better penetrance and preservation of the fluorochromes. We achieved this by introducing the digestion of hyaluronic acid as well as bleaching using milder peroxide on tissues immunolabeled with fluorescently-coupled antibodies after fixation of the organs.

**Table 2:** Comparison of EMOVI to BALANCE and Ce3D protocols.

	hyaluronidase treatment	antibody labelling	bleaching	dehydration	clearing
**EMOVI**	**YES**	**as Ce3D**	**YES** **“aged” solution**	**YES** **isopropanol**	**ECi**
**BALANCE**	**NO**	**as Ce3D**	**YES** **freshly prepared solution**	**YES** **ethanol**	**ECi**
**Ce3D**	**NO**	**in PBS** **FCS** **mouse serum Triton X-100**	**NO**	**not applicable**	**N-methylacetamide** **Histodenz** **Triton X-100** **1-thioglycerol**

We next examined the performance of our EMOVI protocol for labeling, clearing, and volumetric imaging by confocal microscopy for a variety of mouse organs. We observed robust vessel labeling (using anti-CD31 or anti-Lyve-1 antibodies) as well as labeling of MHC-II expressing lymphocytes of lung, lymph nodes, WAT, kidney, brain, liver and heart (**Figure 1C**). Confocal microscopy revealed MHC-II expressing cells with single cell resolution in all organs except for the brain, in which we did not detect MHC-II labeling. Z-stacks of up to 190 µm depth were easily reached (**Figure S3A**), and penetration depth was only limited by the working distance of the 20× objective available to us (about 500 µm for the 20× 0.75 NA objective—see also **Figure 5C** and **Video S3** for the full penetration depth). We also tested the longevity of the fluorescent labeling in EMOVI cleared organs and found that, indeed, clearing with and storage in ECi enabled us to image samples over at least 4 months with little fading of the fluorochromes, as indicated by the same laser settings used (**Figure S3B**). Furthermore, EMOVI cleared organs could be re-used for cryo-sectioning and histology (**Figures S1C, S3C**), with the fluorescence of previously stained antibodies still present (**Figure S3C**).

We hence here describe an easy-to-use, non-toxic, straight-forward, antibody-based tissue immunolabeling and ECi clearing protocol that can be used with a variety of organs with a long-term stability of fluorescently labelled cells.

### Visualization and Quantification of Single Cells in Lung Tissue Using EMOVI

**Figure 2:**
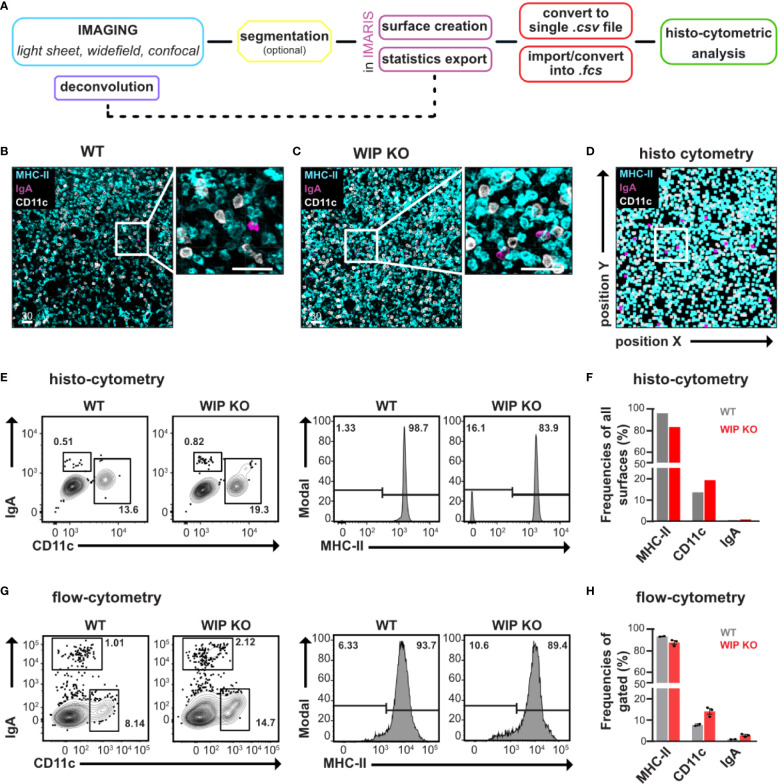
EMOVI of lung tissue demonstrates cellular resolution allowing quantitative analysis. **(A)** Workflow describing our EMOVI approach to detect single cell distribution within tissue. **(B, C)** Lung tissue was stained with the indicated antibodies, cleared with ECi and a 50 µm z-stack imaged by confocal microscopy with single cell resolution (zoom-in panel on the right). Analyzed areas were chosen randomly. **(D)** Statistics for cellular objects were exported into FlowJo and used to quantitatively visualize cellular positioning of the WIP KO lung image in **(C)**. **(E)** Histo-cytometry allows cellular gating of CD11c and IgA expressing populations as well as MHC-II expression in lung tissue of WT and WIP KO mice. **(F)** Bar charts indicate the frequencies of stained cells of all surfaces generated **(G)** Lung tissue of WT and WIP KO mice was enzymatically digested, stained and analyzed by flow-cytometry. CD11c and IgA expressing populations as well as MHC-II expression are shown. **(H)** Bar charts show quantification of the flow-cytometric analysis. Scale bars in µm. Representative images of at least two independent experiments are shown (n = 3 mice per group for flow-cytometric analysis).

Single cell resolution obtained with confocal microscopy enables the quantitative analysis of cells using surface creation and histo-cytometry as previously described (21, 23). We here used a simplified method with optional segmentation to detect single cell distributions within tissue niches (**Figure 2A**). In order to investigate changes in sparse cell populations in lung tissue, we made use of a mouse model of systemic autoimmunity and immunodeficiency (WIP deficient mice, WIP KO). These mice spontaneously develop systemic inflammation including pneumonitis (24). After perfusion of the mouse, the complete lung was removed and lung tissue either used for enzymatic digestion to extract single cells followed by flow-cytometric analysis or fixed for 3D imaging.

Analysis of confocal images of 50 µm thick Z-stacks demonstrated single cell resolution in lung tissue (**Figures 2B, C** and **Figure S4A**). We used 3D surface creation by Imaris to extract statistics of cellular objects, which we then imported into FlowJo using our own “Imaris statistics converter” tool to visualize cellular positioning (**Figure 2D** and **Figure S4B**). We next compared these surface-statistics used for histo-cytometry with statistics of single cells isolated from digested tissue and analyzed by flow-cytometry. Direct comparison of MHC-II, CD11c, and IgA expressing populations obtained by flow-cytometry or histo-cytometry demonstrated comparable patterns of population frequencies (**Figures 2E–H**). Using flow-cytometric analysis, we observed small changes in cell populations in enzymatically digested inflamed lung tissue from WIP KO mice, such as an increased population of CD11c positive cells (15% of gated cells compared to 10% in healthy lung tissue) as well as IgA expressing cells (2% compared to 1% in healthy lung tissue—**Figures 2G, H**). Our histo-cytometric approach demonstrated comparable changes between imaged WT and WIP KO lung tissue (**Figures 2E, F**). From these results we conclude that our EMOVI approach is able to detect even small changes in cell populations of single cells comparable to data obtained by flow-cytometry. At the same time EMOVI maintains the information on spatial localization at cellular level.

### 3D Imaging Followed by Histo-Cytometry Reveals Cell Infiltration of MHC-II Positive Cells in Kidney Glomeruli Containing IgG Deposits

Immune-mediated renal disease leading to kidney malfunction can be caused by immunoglobulin deposits which are commonly detected using thin section imaging. We next employed 3D imaging using EMOVI to investigate immunoglobulin deposits and immune cell infiltration in the kidney of mice prone to develop glomerulonephritis (WIP KO mice) (24).

**Figure 3:**
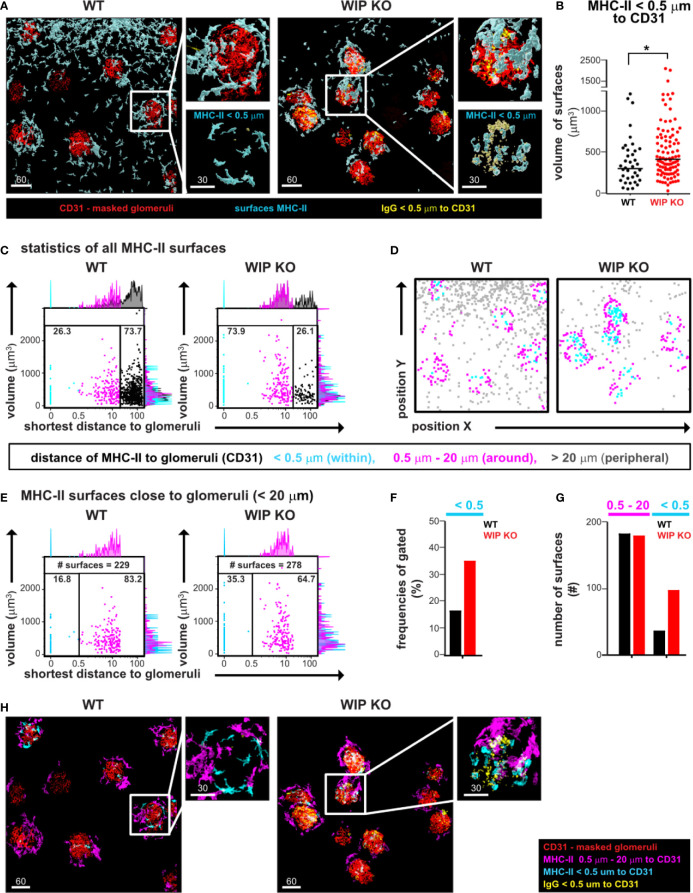
EMOVI followed by histo-cytometry reveals cell infiltration of MHC-II positive cells in kidney glomeruli containing IgG deposits. **(A)** Kidneys from a WT or WIP KO mouse were stained with the indicated antibodies and imaged by confocal microscopy, followed by surface creation using IMARIS. Analyzed areas were chosen randomly. Zoom-in panels show details of glomeruli, MHC-II expressing cells, and IgG deposits. **(B)** Surface volumes of MHC-II infiltrating cells with a distance of <0.5 µm to glomeruli (bar defines the geometric mean; *p = 0.043). **(C, D)** Statistics of total MHC-II surfaces were exported into FlowJo and used to **(C)** quantify the distance of MHC-II surfaces to glomeruli and **(D)** to visualize cellular positioning of gated populations. Populations were defined as shown in the legend. **(E)** MHC-II surfaces with a distance of <20 µm to glomeruli were gated to reveal an increase in MHC-II surfaces within glomeruli in inflamed kidney tissue. Frequencies are quantified in **(F)** and total numbers of surfaces shown in **(G). (H)** Gates defined in histo-cytometry were applied to visualize MHC-II cells surrounding or within glomeruli. Scale bars in µm. Representative images of at least two independent experiments are shown.

We used 1 mm thick vertical sections of WT and WIP KO kidneys, stained them with antibodies against CD31 to reveal vessels and glomeruli, MHC-II to detect antigen-presenting cells, and IgG to visualize immunoglobulin deposit, and cleared them according to our EMOVI protocol. Single-plane overview images by confocal microscopy demonstrated IgG deposits in all visible glomeruli in kidneys of WIP KO mice (**Figure S5A**). We next imaged 100 µm thick Z-stacks of randomly selected areas to detect glomeruli, MHC-II expressing cells as well as IgG deposits (**Figure 3A**, **Figures S5A–C, Video S1**). For analysis, we used 3D surface creation by Imaris to separately mask glomeruli and vessels derived from the CD31 stain (**Figure S5B**). To define cells infiltrating glomeruli or IgG deposits within glomeruli, we next applied shortest distance analysis (distance < 0.5 µm) of surfaces for MHC-II expressing cells and IgG deposits to glomeruli surfaces using Imaris (**Figure 3A** and **Figure S5C**). Quantitative analysis demonstrated an enlarged volume of MHC-II surfaces within inflamed glomeruli of WIP KO mice (**Figure 3B**) as well as an increase in the number of total IgG surfaces but also in larger volume IgG surfaces (**Figure S5E**). Of note, a 7 µm thick section cropped from the complete image showed only little IgG deposits and no cellular structure of MHC-II expressing cells in glomeruli (**Figure S5D**) demonstrating the limitation of traditional thin section 2D imaging. EMOVI thus permitted volumetric multiplex imaging demonstrating IgG deposits and immune cell infiltration as measure of disease activity in nephritic kidneys.

Next, we imported all surface statistics into FlowJo to quantify frequencies and further characterize MHC-II expressing cells in WT and WIP KO tissue. We noted three different populations in relation to the distance to the closest glomeruli: a population within glomeruli (<0.5 µm, cyan), an intermediate population (0.5–20 µm, magenta) and a peripheral population with the longest distance to glomeruli (>20 µm, gray populations in the histograms and gates of **Figure 3C**). Concomitant to the corresponding images (**Figure 3A**), we found the total number of MHC-II surfaces with the longest distance to glomeruli increased in the analyzed WT tissue (73.7% of MHC-II surfaces compared to 26.1% of surfaces in WIP KO tissue, **Figure 3C**). Visualizing those gated populations by cellular positioning in X and Y using FlowJo allowed us to define the intermediate population as MHC-II surfaces surrounding the glomeruli with a distance between 0.5 and to 20 µm (**Figures 3C, D**). To accurately determine the frequency of MHC-II surfaces within glomeruli, we excluded MHC-II surfaces in the periphery by gating on all MHC-II surfaces with a distance smaller than 20 µm to glomeruli (**Figure 3E**) and found similar numbers of these surfaces between the WT (229 surfaces) and WIP KO (278 surfaces) tissues (**Figures 3E, G**). Having normalized to a similar cell number like this, we found the MHC-II surfaces within WIP KO glomeruli to be increased [35% in WIP KO glomeruli compared to 16.8% in WT glomeruli (**Figures 3E–G**)]. We also used the FlowJo gates to define masks in Imaris for visual representation of the MHC-II expressing cells surrounding the glomeruli (**Figure 3H**, **Video S1**). We conclude that the quantitative visualization of cellular positioning using FlowJo allowed us to define specific populations of MHC-II expressing cells and to more accurately determine and visualize the increase of MHC-II surfaces within glomeruli of nephritic kidneys.

### EMOVI Allows Imaging From Large Volume Overviews to Single Cell Resolution and the Comparison of Different Microscopes

**Figure 4:**
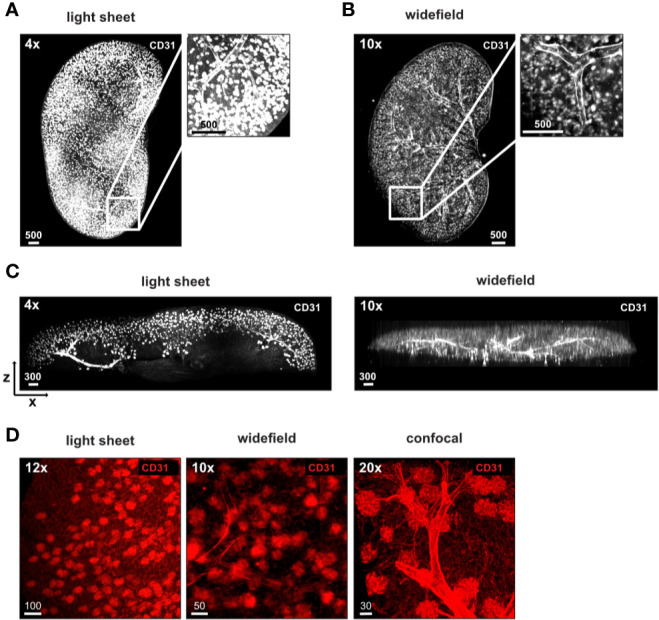
Stable antibody labeling allows usage of different microscopes. **(A–D)** Representative images of antibody stained, ECi cleared half kidneys acquired using a **(A)** light sheet fluorescence microscope, or a **(B)** widefield microscope with magnifications and staining as indicated. **(C)** X and Z displays demonstrate a high resolution in Z of images acquired by light sheet microscopy (750 µm section) and lower resolution in Z of images acquired by widefield microscopy (complete image). **(D)** Higher magnification of glomeruli demonstrates differences in resolution of glomeruli between all three microscopes used. Scale bars in µm. Representative images of two independent experiments are shown.

We next aimed at transitioning from a general overview of the complete tissue to the analysis of specific regions within the tissue at higher resolution thereby comparing a light sheet fluorescent microscope, a widefield microscope and a confocal microscope. We first analyzed vasculature and glomeruli of anti-CD31 labeled kidneys. To obtain an overview of the tissue, we imaged half a kidney with a light sheet and a widefield microscope (**Figures 4A, B**). Both types of microscopes resolved vessels and kidney glomeruli in X and Y (**Figures 4A–D**). Images of the light sheet and widefield microscopes demonstrated evenly stained glomeruli throughout half the kidney analyzed (**Figures 4A–C**, **Video S2**). The size of datasets acquired was similar between both imaging modalities (94 GB of the light sheet image compared to 85 GB of the widefield image, **Table 3**). Due to the mode of illumination, the image acquired with the widefield microscope showed a limited resolution in Z compared to the light sheet microscope, albeit similar voxel size (**Figure 4C**, **Table 3**). A higher magnification revealed a more detailed structure of the kidney glomeruli also with the widefield microscope (**Figure 4D**), indicating a good signal-to-background ratio of the staining. Data comparing dimensions of images in **Figure 4** as well as data size generated, imaging time, resolution, and normalized imaging time per sequence and volume can be found in **Table 3**.

**Table 3:** Details of images in **Figure 4**.

kidney	size in mm (x,y,z)	acquisition time (HH : MM:SS.ms)	file size(MB)	size in µm(x,y,z)	voxel size in nm (x,y,z)	voxel (x,y,z)	number of channels/sequences	time (s) per sequence and 1nL volume [100^3^ µm^3^]
**Figure 4A** **(light sheet)**	9.3, 9.2, 4.6	30:34:29.000	9,4110	9,251, 9,199, 4,590	1,625, 1,625, 2,998	5,693, 5,661, 1,531	1/1	0.282
**Figure 4B** **(widefield)**	8.4, 6.1, 1.4	00:02:20.970	8,9069	8,410, 6,060, 1,370	642.63, 642.63, 3,790	13,089, 9,425, 361	1/1	0.002
**Figure 4****C** **same as** **Figures 4A**, **B**								
**Figure 4D** **(light sheet)**	1.1, 1.1, 1.2	00:10:02.000	12,448	1,109.3, 1,109.3, 1,167	542, 542, 3,000	2,048, 2,048, 389	4/4	0.105
**Figure 4D** **(widefield)cropped** **Figure 4B**	2.9, 2.9, 1.4	na	14,003	2,931, 2,867, 1,370	642.63, 642.63, 3,790	4,561, 4,459, 361	1/1	na
**Figure 4D** **(confocal)**	0.5, 0.5, 0.4	01:49:55.517	2,894	455.88, 455.88, 357.92	223, 223, 1,040	2,048, 2,048, 345	2/2	44.333

**Figure 5:**
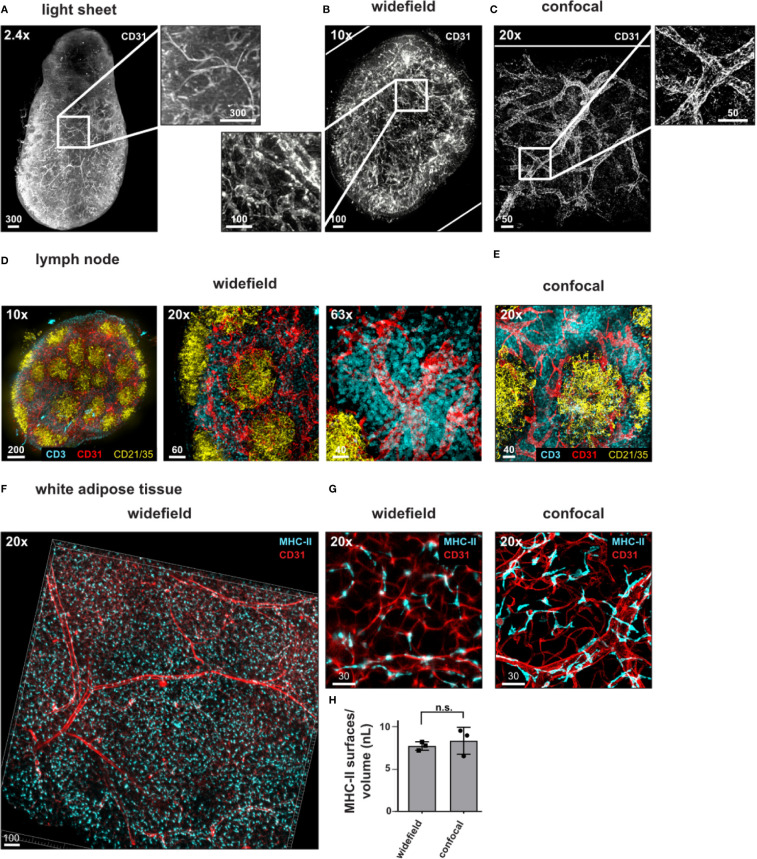
Widefield microscopy with real-time computational clearing for rapid imaging of single cells. **(A–D)** Representative images of antibody stained, ECi cleared lymph nodes acquired using a **(A)** light sheet fluorescence microscope, **(B, D)** widefield microscope or **(C, E)** confocal microscope with magnifications and staining as indicated. Magnified areas indicated with white boxes. **(F, G)** Widefield microscopy using a 20× objective reveals vessels and MHC-II expressing cells in white adipose tissue. **(G)** Widefield image compared to confocal image acquired with a 20× objective (zoom factor of 1) with similar magnification. **(H)** Analyzed areas were chosen randomly. Mean values +/− SD of three images each is shown. n.s = not significant. Scale bars in µm. White rims indicate image borders. Identical images of lymph nodes are displayed in **Figure S5A, B**. Representative images of at least three independent experiments are shown.

Compared to the kidney, lymph nodes are small structures, which are feasible to image as a whole by confocal microscopy, albeit in a time-consuming process (14, 21, 25, 26). We next wanted to explore the possibility of using the widefield microscope as a faster alternative to transit from a general overview of the complete tissue to the analysis of specific regions within the tissue at higher resolution. We obtained images of the lymph node vasculature stained with anti-CD31 throughout the complete EMOVI treated lymph node with the light sheet and the widefield microscope in reasonable time [10 min for acquisition with the light sheet microscope (six sequences) and 9 min for acquisition with the widefield microscope (four sequences)] (**Figures 5A, B** and **Table 4**). As comparison, we acquired only a small part but the whole depth (500 µm) of the lymph node by confocal microscopy, which was the maximum working distance for the 20× objective used (acquisition time = 3.5 h for three channels in two sequences, **Figure 5C**, **Table 4** and **Video S3**). Using the widefield microscope, we went from the overview of the complete lymph node (**Figure 5D** and **Video S4**) with a 10× objective to a further increase in magnification using a 20× as well as a 63× objective. Higher magnification revealed smaller structures with cellular resolution (**Figure 5D**), comparable to the confocal image obtained with a 20× objective and similar voxel size (**Figure 5E**, **Table 4**). Image processing to obtain cellular positioning and single cell detection was possible with images obtained by the widefield microscope (see histo-cytometry, **Figures S6A, B**). However, a limited resolution in Z made the surface creation of densely populated regions (such as the CD3 area in the lymph node) difficult (**Figure S6B**). Using the widefield microscope, we also obtained cellular resolution of MHC-II stained cells in EMOVI treated kidneys (**Figure S6C**) and white adipose tissue (WAT) (**Figures 5F, G** and **Video S5**). Quantifying MHC-II expressing cells in images acquired by confocal microscopy or widefield microscopy of white adipose tissue demonstrated similar numbers of MHC-II expressing surfaces normalized to the volume of the tissue (**Figures 5G, H**). Of note, normalizing the imaging time to the number of sequences and a volume of 1 nanolitre (nl), we found that imaging with the 20× objective using the widefield microscope was about 100 times faster compared to imaging with the 20× objective using the confocal microscope at similar voxel size [0.225 s compared to 20.53 s per sequence and 1 nl for the lymph node (**Table 4**) and 0.33 s compared to 36.34 s per sequence and 1 nl for the WAT (**Table 4**) using the widefield microscope compared to the confocal microscope respectively]. Data comparing dimensions of images in **Figure 5** as well as data size generated, imaging time, resolution and normalized imaging time per sequence and volume can be found in **Table 4**. An overview of tissues tested as an indication for feasibility especially comparing widefield and confocal microscopy is depicted in **Table 5**.

**Table 4:** Details of images in **Figure 5**.

lymph node	size in mm (x,y,z)	acquisition time (HH : MM:SS.ms)	file size(MB)	size in µm(x,y,z)	voxel size in nm (x,y,z)	voxel (x,y,z)	number of channels/sequences	time (s) per sequence and 1nL volume [100^3^ µm^3^]
**Figure 5A** **(light sheet)**	5.5, 5.5, 1.3	00:09:57.000	6,000	5,546.0, 5,546.0, 1,250	2,708, 2,708, 10,000	2,048, 2,048, 125	6/6	0.069
**Figure 5B** **(widefield)**	2.5, 2.5, 0.6	00:08:48.142	1,9596	2,510.86, 2,506.32, 616.41	3,880, 3,873, 163	647, 647, 3,805	4/4	0.034
**Figure 5C** **(confocal)**	0.6, 0.6, 0.5	03:28:43.829	1,2104.8	581.25, 581.25, 499.42	2,048, 2,048, 481	284, 284, 1,040	3/2	37.11
**Figure 5D** **(10× widefield)same as** **Figure 5B**	2.5, 2.5, 0.6	00:08:48.142	19,596	2,510.86, 2,506.32, 616.41	3,880, 3,873, 163	647, 647, 3,805	4/4	0.034
**Figure 5D** **(20× widefield)**	0.7, 0.7, 0.6	00:03:44.302	7,986	66,2.51, 6,62.51, 568.12	2,048, 2,048, 238	324, 324, 2,397	4/4	0.225
**Figure 5D** **(63× widefield)**	0.2, 0.2, 0.1	00:05:05.494	1,1476	210.32, 210.32, 144.02	2,048, 2,048, 342	103, 103, 422	4/4	11.99
**Figure 5E****(20× confocal)**	0.6, 0.6, 0.3	02:32:07.545	1,0654	581.25, 581.25, 263.24	2,048, 2,048, 254	284, 284, 1,040	5/5	20.53
**WAT**	**size in mm (x,y,z)**	**acquisition time (HH : MM:SS.ms)**	**file size** **(MB)**	**size in um** **(x,y,z)**	**voxel size in nm (x,y,z)**	**voxel (x,y,z)**	**number of channels/** **sequences**	**time (s) per sequence and 1nL volume [100^3^ um^3^]**
**Figures 5F, G****(20× widefield)**	1.8, 1.9, 1.0	01:16:27.652	11,2182	1,848.02, 1,856.05, 1,009.20	321, 321, 2397	5752, 5,777, 422	4/4	0.33
**Figure 5G****(20× confocal)**	0.6, 0.6, 0.1	01:25:35.556	4,530	581.25, 581.25, 139.42	284, 284, 1,040	2,048, 2,048, 135	4/3	36.34

**Table 5:** Overview of organs tested with indicated microscopes.

	lung	lymph node	WAT	kidney	brain	liver	heart
**light sheet**	**YES**	**YES**	**n.d**.	**YES**	**n.d**.	**n.d**.	**n.d**.
single cell resolution	**YES**	**NO**	**n.d**.	**NO**	**n.d**.	**n.d**.	**n.d**.
**widefield**	**NO**	**YES**	**YES**	**YES**	**YES**	**NO**	**NO**
single cell resolution	**NO**	**YES**	**YES**	**YES**	**NO**	**NO**	**NO**
**confocal**	**YES**	**YES**	**YES**	**YES**	**YES**	**YES**	**YES**
single cell resolution	**YES**	**YES**	**YES**	**YES**	**YES**	**YES**	**YES**

These results demonstrate that EMOVI allows imaging from whole organ overviews to single cell resolution. We found that the widefield microscope can be used to obtain a 3D overview of large organs such as the kidney and smaller organs such as the lymph node in a short time with reasonable resolution. This information can be used to define specific regions of interest for further in-depth analysis using the widefield or the confocal microscope, depending on the information needed.

## Discussion

Spatial information of cells in the context of their microenvironment is crucial to understand the complexity of inflammatory processes. Volumetric imaging of cleared large tissue pieces or whole organs can provide this information and is a valuable alternative to traditional immunofluorescence or histology of thin tissue sections. We here describe EMOVI, a simplified, cost-effective and non-harmful whole-mount imaging approach that enabled and preserved multiplexed antibody-based immunolabeling on fixed organs, allowing long-term storage of samples and flexibility in microscope usage.

Published protocols often use i.v. injection of antibodies shortly before or after sacrificing mice to improve antibody penetration and hence reduce staining time (13, 17, 19). Although this is the fastest way to obtain a homogenous staining, we decided not to use i.v. injection in order to keep EMOVI flexible and allow multiplexing of antibodies. Other recently published protocols demonstrated immunolabelling to detect structural components, neurons or vasculature in fixed organs (4, 27–30); however, reports describing the detection of immune cells without the use of transgenic animals expressing cell-type specific fluorescent reporters remain rare (14, 21, 26). Our results demonstrated that EMOVI allows multiplexed antibody-based immunolabeling of immune cell populations in whole organs or large tissue pieces after fixation with the advantage of preserving fluorescence over several months.

Crucial changes to currently published protocols include the digestion of parts of the ECM by hyaluronidase, the use of isopropanol for dehydration and reducing light scattering by organ-bleaching using peroxide after immunolabeling of fixed tissues. In combination, these changes improved antibody penetrance and minimized light scattering and autofluorescence caused by differences in the macromolecular content (lipids, ECM) of the tissues used (29, 31). We found the digestion of hyaluronic acid more helpful than the often used but time-consuming de-lipidation and de-calcification steps by highly toxic chemicals [reviewed in (15)]. However, EMOVI uses washing solution containing low concentrations of Triton-X 100, which further improves tissue penetrance along with de-lipidation (14). In addition to hyaluronidase treatment, we here often refrained from staining complete organs and instead used around 1mm thick organ slices or pieces to further improve antibody penetration into fixed organs, as has also been recommended by others (4, 14, 26, 32). We found this type of sectioning crucial especially if aiming at homogenous staining for immune cell populations in organs such as the kidney, liver and heart. Thick tissue slices also improve the handling and mounting of organs for imaging, reduced imaging time while at the same time maintain sufficient spatial information. We however would like to point out that sectioning always introduces a sampling bias that needs to be considered and corrected for, e.g. through random selection of multiple sections and areas (33).

Applying EMOVI allowed us to detect sparse cell populations during inflammatory processes such as pneumonitis or glomerulonephritis as well as revealing cellular positioning and enabling quantitative analysis using FlowJo. Lung tissue is in our hands a relatively easy tissue to clear and quantify, as the spongy nature of the organ allows for fast and easy antibody penetration and single cells are distributed evenly throughout the tissue. Our quantitative analysis using surface statistics and histo-cytometry detected a higher infiltration of a rare IgA as well as CD11c expressing cell population in inflamed mouse tissue which was comparable to results obtained by flow-cytometry of digested lung tissue. By increasing the numbers of antibodies used for staining, EMOVI hence has the potential to be employed for in-depth analysis of cell population changes in lung tissue due to inflammation, infection or tumor formation while at the same time revealing spatial information of the microenvironment. We suggest that the image quality also allows quantification of cells using elaborate, recently published algorithms (26) in addition to the straight forward analysis shown here.

In contrast to lung tissue, kidney tissue needs hyaluronidase treatment and bleaching to gain maximum transparency and light penetration. As such, hallmarks of kidney malfunction, like the total number of glomeruli, their volume, the depletion of glomerular epithelial cells (podocytes), antibody deposition as well as immune cell infiltration are traditionally analyzed by thin section imaging in 2D. This 2D approach has been shown to be prone to errors, missing minor differences of disease severity throughout the kidney by introducing a sampling bias [reviewed in (34)]. By looking at a 7 µm thick optical section, we also found only little IgG deposits and no cellular structure of MHC-II expressing cells in glomeruli, hence making it difficult to determine cell numbers and to address distances of MHC-II expressing cells to glomeruli, again demonstrating the limitations of this 2D approach. To overcome these issues, 3D imaging of the cleared kidney using LSFM (19) or imaging of the cleared kidney by slicing and confocal or 2-photon microscopy (17, 35, 36) has been developed recently. Using EMOVI, we here demonstrated a homogenous staining of CD31 positive endothelial cells throughout half a kidney (about 1.5 mm depth) after fixation, which would also allow determining glomeruli counts and volume using algorithms and analysis tools described before (17, 19).

Using our EMOVI approach, we detected MHC-II expressing cells scattered throughout the kidney during homeostatic conditions and infiltrating kidney glomeruli containing IgG deposits during inflammatory conditions. The combination of surface creation in Imaris and population gating in FlowJo using histo-cytometry allowed us to accurately define and visualize MHC-II expressing cells surrounding or infiltrating glomeruli of nephritic kidneys. While we found MHC-II expressing cells surrounding glomeruli in similar numbers also in WT kidneys, MHC-II expressing cells within glomeruli mainly occurred in glomeruli containing IgG deposits. We hypothesize that MHC-II expressing cells surrounding the glomeruli in a homeostatic condition might be tissue-resident macrophages (17, 37), whereas MHC-II expressing cells infiltrating inflamed glomeruli might be monocytes (38, 39) that potentially detect immune-complexes by their Fc-receptors (37). Combining 3D imaging and histo-cytometry, we here demonstrate the power and possibilities of a detailed analysis of MHC-II expressing immune cells infiltrating into inflamed glomeruli containing IgG deposits.

We further compared light sheet fluorescence microscopy, to get an overview of the complete tissues stained; widefield microscopy, to test both, an overview as well as higher resolution; and confocal microscopy, to interrogate specific cellular localization and interaction. Our aim was not to compare these microscopes in detail but to give an indication of imaging speed, resolution, data size generated and feasibility. The light sheet fluorescence microscope has been widely used to study the distribution of vessels and nerves in large organs such as the kidney, heart, brain and even whole animals (13, 19, 29, 30, 40, 41). Light sheet microscopy has a higher resolution in Z compared to widefield microscopy, but this also comes at the expense of time. Nevertheless, we found it more challenging to image smaller organs such as lymph nodes or tissue sections using the light sheet microscope due to the limitations of the sample holder. Furthermore, signal detection using the light sheet microscope works best for longer-wavelength emission spectra, demanding fluorochromes tailored to this need. We here avoided adapting fluorochromes accordingly in order to use antibodies coupled to fluorescent dyes widely used for flow-cytometry or confocal microscopy to keep EMOVI as simple and flexible as possible. We here mainly focused on widefield microscopy with real-time computational clearing as a novel and potentially faster imaging modality for overview images compared to light sheet microscopy and for cellular resolution compared to confocal microscopy.

Imaging immune cells in large-volume samples by confocal microscopy is a time-consuming process but provides a maximum of cellular resolution (14, 21, 26). To increase imaging speed, our results suggest that the widefield microscope with real-time computational clearing is a valuable and much faster alternative to confocal microscopy. We here used widefield microscopy with real-time computational clearing to generate overview images of whole organs in short time. These overview images can subsequently be used to detect and quantify rare cell populations or loosely spread cells throughout for example whole lymph nodes, half a kidney or large pieces of fat tissue. However, we found that a limited resolution in Z due to the nature of the method (mode of illumination and image acquisition) made statistical analysis of densely populated regions (such as T cells in the lymph node) or the resolution of smaller vessel deep in the tissue difficult in the total volume imaged and requires more work in future. In addition, the widefield microscope is susceptible to tissue autofluorescence and we found acquisition of single cells in lung, liver and heart challenging. Hence, although imaging thin tissue sections of these tissues by widefield microscopy should be feasible, volumetric imaging of lung, liver and heart using the widefield microscope needs further reduction of tissue autofluorescence. We found that more aggressive clearing methods, such as for example Ce3D (14), or bleaching with a freshly prepared peroxide solution (13) led to better light penetration and less light scattering, but at the same time reduced fluorescence intensity and hence signal-to-background ratio. Further optimization of the protocols is needed in order to be able to image those organs with cellular resolution using the widefield microscope.

Widefield microscopy can generate large datasets in short time. In addition, images obtained by widefield microscopy require deconvolution to regain spatial resolution. Thus, when imaging with a widefield microscope, data storage and processing power of the analysis computer need to be considered.

Spatial information of cells in their tissue microenvironment is necessary to understand the complexity of inflammatory or pathophysiological processes. We here present EMOVI and demonstrate possible applications of this 3D imaging approach: The use with multiple organs, possibilities of in depth analysis, comparing different ways of imaging and introducing widefield microscopy. By facilitating flexibility in an organ, fluorochrome and microscopy usage, EMOVI hence has the potential to make tissue clearing and volumetric imaging an interesting tool for a broad audience. Our manuscript might add to the information needed to investigate *e.g.* tissue damage during viral infections, the tumor microenvironment, neuroinflammation, and many more processes that demand spatial information of tissues and cells within.

## Material and Methods

### Mice

*Wipf1^-/-^* mice (42) were a kind gift from Raif Geha (Boston’s Children Hospital, Boston, USA); BALB/c WT mice were either littermates or bought from Charles River. All animal experiments were performed according to local guidelines (Regierung von Oberbayern, Munich, Germany).

### EMOVI Sample Preparation

Mice were sacrificed by an overdose of isoflurane and immediately transcardially perfused with PBS or 2.5 mM EDTA in PBS. Harvested tissues were fixed in IC Fixation Buffer (eBioscience) for 1 h at room temperature (RT) and in 25% (v/v) fixation buffer in PBS overnight in 4 °C. All following incubations were performed in 2 ml tubes with gentle agitation.

#### Digestion (Optional)

Samples were washed in PBS with 1% (v/v) FCS, 1% (v/v) normal mouse serum (Jackson Immunoresearch) and 0.3% (v/v) Triton X-100 (‘Blocking Buffer’) for 1 h at 37 °C and incubated with 300 µg/ml hyaluronidase (from bovine testes, Type IV-S; Sigma Aldrich) for 2 h at 37 °C. After digestion tissues were washed again for 1 h to remove the remaining enzyme.

#### Antibody Staining

Tissues were incubated in Blocking Buffer overnight for at least 12 h at 37 °C and stained with directly fluorochrome conjugated, primary antibodies, diluted 1:100 to 1:300 in Blocking Buffer (antigen and tissue specific), for 72 to 96 h at 37 °C. The following steps were performed protected from light.

#### Dehydration and Clearing

Stained samples were washed in PBS with 0.2% (v/v) Triton X-100 and 0.5% (v/v) 1-thioglycerol for 16 h at 37 °C. For tissues with high intrinsic autofluorescence the washing solution was renewed twice. Tissues were then dehydrated with ascending dilutions of 30, 50 and 70% (v/v) isopropanol (pH ~9) for 1 h each and finally with 100% (v/v) isopropanol for 2 h at 4 °C.

Tissues rich in intrinsic pigments were in addition bleached with a slightly ‘aged’ (at least one week) solution of 5% (v/v) hydrogen peroxide and 5% (v/v) DMSO in isopropanol for 4 h at 4 °C and then again fully dehydrated in 100% (v/v) isopropanol for 1 h at 4 °C.

To avoid ethyl cinnamate (ECi)-mediated freezing damage the samples were allowed to warm up to RT before undiluted ECi was applied. The precise clearing time was sample-dependent but did not exceed 1 h and could be readily assessed by visual inspection. Careful handling of ECi is advised, as the odour of ECi is perceived as very intense by some. In addition, ECi is a mild organic solvent which is known for dissolving glue of imaging chambers or isolation rings of objectives and hence material used with ECi needs to be tested for compatibility [an overview of compatibility with imaging chambers can be found in (20), compatible combinations of materials in (43)]. The samples were stored in the dark at RT in ECi in 1.5 ml Eppendorf tubes, which are compatible with ECi for long-term storage.

### Alternative Clearing Protocols

Tissues were collected and fixed as described for EMOVI.

#### BALANCE

The BALANCE clearing protocol was adapted for post-fixation antibody labelling but otherwise performed as previously described (13). Samples were blocked and stained as described for EMOVI. Tissues were dehydrated with ascending dilutions of 30, 50, 70% (v/v) and twice with 100% (v/v) high-grade ethanol (pH ~9) for at least 4 h each at 4 °C. Samples were bleached with a freshly prepared solution of 5% (v/v) hydrogen peroxide and 5% (v/v) DMSO in ethanol for 4 h at 4 °C and then again fully dehydrated in 100% (v/v) ethanol for 1 h at 4 °C. Clearing was performed as detailed for EMOVI.

#### Ce3D

Ce3D clearing was performed as previously described (14). Samples were washed three times in PBS with 0.2% (v/v) Triton X-100 and 0.5% (v/v) 1-thioglycerol (‘Washing Buffer’) for 30–60 min at 37 °C. Blocking and staining were performed as described for EMOVI. Subsequently tissues were treated with Washing Buffer for 8 h at 37 °C and again for 24 to 36 h at RT. The Washing Buffer was replaced every 10–14 h. Washing Buffer was then carefully removed with precision wipes, and tissues were submerged in freshly prepared Ce3D clearing solution containing 22% (v/v) N-methylacetamide, 0.8 g/ml Histodenz, 0.1% (v/v) Triton X-100 and 0.5% (v/v) 1-thioglycerol in PBS. Samples were incubated for at least 24 h at RT and imaged in clearing solution.

### Analysis of Tissue Morphology

#### Sample Preparation

Cleared samples were incubated in descending dilutions of 100, 100, 70, 50, and 30 (v/v) isopropanol (pH ~9) and then Blocking Buffer for 1 h each at RT to fully remove ECi and carefully rehydrate the tissue.

Samples were incubated in 30% (w/v) sucrose solution ON at 4 °C, embedded in O.C.T. compound and frozen on dry ice. 10 µm sections were cut on a Leica CM3050 S Research Cryostat and stored at −80 °C. Before further processing slides were dried in a silica chamber for 10 min at RT.

#### Immunofluorescence

Sections of recycled tissues were mounted with Fluoromount-G (Southern Biotech) and sealed with clear nail polish.

#### Histology

Samples were incubated in 0.1% (w/v) Mayer’s haematoxylin solution for 10 min and developed under cold running tap water (slightly alkaline) for 5 min. Subsequently, slides were submerged several times in 0.5% (w/v) eosin solution, briefly washed in distilled water and submerged in ascending ethanol dilutions of 50 and 70% EtOH several times. Next slides were incubated in 95 and 100% EtOH for 30 s or 1 min, respectively. Samples were then submerged in xylene several times and mounted with Roti-Histokitt II mounting medium (Carl Roth).

### Image Acquisition

Cleared samples were mounted in glass coverslip bottom slides (ibidi) submerged in ECi and weighed down with an additional coverslip to inhibit movement during imaging. For analysis of immune cells in the lung, MHC-II expressing cells in kidneys and WAT randomly chosen areas were selected. After image acquisitions, sample and clearing agent were removed from the chamber to conserve the slide for reuse.

Confocal imaging was performed on an inverted Leica TCS SP8 confocal microscope with White Light Laser and HyD photodetectors using a HC PL APO CS2 20×/0.75 IMM objective (zoom factor of 1). The following acquisition settings were applied: 2,048 × 2,048 logistical size, 400 Hz scan speed, bidirectional scan mode, 4× line average, system optimized z-step size, sequential scans between frames, active time gating and a reduced pinhole for samples with high autofluorescence. The bit depth can be increased to the highest setting that still enables signal saturation with reasonable excitation laser power.

Volumetric widefield images were acquired using a Leica THUNDER Imager 3D Cell Culture equipped with a DFT51011 multi-band cube in combination with a fast external filter-wheel, SPECTRA X or LED8 multi-LED light source and a Leica DFC9000 GTC camera.

The following objectives were used: N PLAN 5×/0.12 DRY, HC PL FLUOTAR 10×/0.32 DRY, HC PL FLUOTAR L 20×/0.40 DRY and HC PL FLUOTAR 63×/1.4–0.6 OIL.

Light sheet fluorescence images were captured on an UltraMicroscope II (Miltenyi Biotec). Depending on the fluorophores in the sample the laser lines 405, 488, 561, and 640 nm were used. Detection was done with dynamic horizontal focus using a 4× objective (Miltenyi Biotec MI PLAN 4×/0.35 NA) or 12× objective (Miltenyi Biotec MI PLAN 12×/0.53 NA) coupled to a Super Plan Unit with the emission filters 460/40, 525/50, 595/40, 620/60 and 680/30. For measurements with the 4× objective, a re-magnification of 0.6× was chosen resulting in an overall magnification of 2.4×. Exposure times and laser powers were adapted to each sample depending on the fluorescent intensity. The light sheet width was kept at 80% width for kidney and 90% width for lymph nodes.

Histological stainings were imaged with a Zeiss AXIOS Imager.M2 equipped with a Zeiss AxiosCam Mrc 5 color camera using Zeiss EC ”Plan-Neofluar” 10×/0.30 M27 and EC ”Plan-Neofluar” 20×/0.50 M27 objectives.

### Data Processing and Analysis

Time requirements for the different imaging modalities were normalized to the number of acquisition sequences and the imaged sample volume according to the following equation:

$$\frac{acquisition\;time\;\left[s\right]}{\#\;of\;sequence\;\times \;aquired\;volume\;\left(x*y*z\right)\;\left[nL\right]}.$$

Images were deconvolved using the LIGHTNING module in LAS X (Leica, https://www.leica-microsystems.com/science-lab/how-to-extract-image-information-by-adaptive-deconvolution/) and widefield images were furthermore processed with the Large Volume Computational Clearing module (LVCC, Leica). Computational Clearing is an opto-digital technology that removes the typical “blur”/”haze” of wide-field images. For that, it automatically takes all relevant optical parameters into account thus maintaining the real local dimensions of the sample. It allows widefield imaging into thick 3D specimens. LVCC parameters were automatically selected based on the respective objective used for imaging. Further Data Processing was performed on a Z640 Workstation (HP; Win10 Enterprise 64-bit; Intel Xeon CPU E5-2650 v3 @ 2.30GHz; 32.0 GB RAM; NVIDIA Quadro K2200 4 GB GDDR5 (DirectX 12.0)).

Contrast adjustment for display purposes and image analysis was performed using Imaris (Bitplane) version 9.5. We used the ‘Surface’ and ‘Spot Creation Wizard’s in Imaris (Bitlane) to translate fluorescence data into volumetric, representative surfaces or point-like spots. For each object, a variety of parameters is calculated. For surfaces, these parameters include for example the position (x, y, z), volume, sphericity, median fluorescence intensities for all channels, volume and the distance to the closest object of defined surfaces/spots. For the analysis of the distance of MHC-II cells to glomeruli, we asked for the distance of MHC-II surfaces to the closest glomeruli surface (“shortest distance”). We next exported selected parameters into FlowJo, analyzed the distribution of MHC-II cells towards their nearest glomerulus as dot plots and histograms, and defined populations according to their distribution (visible separate peaks or populations). Defined populations were verified by looking at the cellular positioning using the X and Y parameters in FlowJo dot plots.

All statistics relevant for analysis were exported as a collection of csv files and subsequently edited and concatenated into a single summary file with our open-access standalone Python application [https://gitlab.com/kepplerlab/imaris_statistics_converter; incorporates ‘pandas’ module (44, 45)] for compatibility with further analysis in FlowJo (BD Biosciences).

For comparative analysis of signal and background fluorescence intensities, we acquired overviews with identical imaging settings at 100 µm depth in z from the first frame filling image plane. Using Fiji (ImageJ) (46, 47) we measured the mean pixel grey values of marked areas of defined signal (glomeruli) or background, at 10 randomly selected positions each in the stitched but otherwise unprocessed images, to calculate the average signal or background intensity, respectively.

Descriptive size and shape analysis of glomeruli structures in images of histological stains was performed using Fiji (ImageJ) (46, 47). Radom areas throughout the entire section were marked with the elliptical selections tool and area and roundness (inverse aspect ratio) of the selections was measured.

### Single Cell Isolation

Lungs of perfused mice were harvested. Half of the tissue was used for EMOVI treatment (*described above*) while the other lobe was rinsed with PBS and placed in lymphocyte medium (RPMI 1640, 10% FCS, 10 mM HEPES, 100 U/ml Penicillin-Streptomycin, 50 µM 2-mercaptoethanol) on ice.

The tissue was transferred to a 100-µm cell strainer in a 6-well plate and minced finely. Lungs were digested in 5 ml of 1 mg/ml collagenase Type I (Gibco) and 0.5 mg/ml DNAse Type I (Roche) in prewarmed HBSS with 10% FCS for 45 min at 37 °C on an interval shaker (30 s every 3 min) with occasional meshing of the tissue. The suspension was subsequently filtered through a 70-µm cell strainer and further depleted for red blood cells (RBCs).

### Flow Cytometry

Single-cell suspensions were incubated with anti-CD16/32 and anti-CD16.2 antibodies in fluorescence-activated cell sorting (FACS) buffer (PBS, 2% FCS) to block Fc receptors and stained with the appropriate combination of the following antibodies: CD11b, CD11c, CD19, CD31/PECAM-1, CD4, CD45R/B220, IgA and MHC-II. Cells were acquired on LSRFortessa and analysed with FlowJo (both BD Biosciences*)*.

### Statistical Analysis

All statistical calculations were performed using Microsoft Excel or GraphPad Prism 7. Analysis of differences of signal and background intensities between methods (**Figure S1B, S2C**) was performed using ordinary two-way ANOVA with Tukey correction for multiple comparisons. Glomeruli size and shape differences (**Figure S1D**) were assessed using unpaired, two-tailed t tests. The comparison of MHC-II surface volumes (**Figure 3B**) was evaluated using the two-tailed Mann–Whitney U test (for samples with unequal distribution). Differences between groups in **Figure 5H** were assessed using the unpaired two-tailed Welch’s t test. Differences are indicated with * for p < 0.05, ** for p < 0.01, *** for p < 0.0001 or n.s. for not significant.

## Reagents or Resources

**Table:** 

REAGENT or RESOURCE	SOURCE	IDENTIFIER
**Antibodies for E-MOVI**		
AF594 anti-CD11c (N418)	BioLegend	Cat# 117346; AB_2563323
AF594 anti-CD21/35 (7E9)	BioLegend	Cat# 123426; AB_2632698
BV421 anti-CD3ϵ (145-2C11)	BioLegend	Cat# 100335; AB_10898314
AF594 anti-CD31/PECAM-1 (MEC13.3)	BioLegend	Cat# 102520; AB_2563319
AF647 anti-CD31/PECAM-1 (MEC13.3)	BioLegend	Cat# 102516; AB_2161029
AF555 anti-IgA (polyclonal)	Southern Biotech	Cat# 1040-32; AB_2794378
AF594 anti-IgG (polyclonal)	Thermo Fisher	Cat# A-11005; AB_2534073
eF660 anti-LYVE-1 (ALY7)	Thermo Fisher	Cat# 50-0443-82; AB_10597449
AF488 anti-MHCII (I-A/I-E) (M5/114.15.2)	BioLegend	Cat# 107616; AB_493523
AF532 anti-MHCII (I-A/I-E) (M5/114.15.2)	Thermo Fisher	Cat# 58-5321-82; AB_2811913
**Antibodies for flow cytometry**		
PerCPCy5.5 anti-CD11c (N418)	BioLegend	Cat# 117328; AB_2129641
purified anti-CD16.2 (9E9)	BioLegend	Cat# 149502; AB_2565302
purified anti-CD16/32 (2.4G2)	BD Biosciences	Cat# 553140; AB_394655
BUV395 anti-CD19 (1D3)	BD Biosciences	Cat# 563557; AB_2722495
AF647 anti-CD31/PECAM-1 (MEC13.3)	BioLegend	Cat# 102516; AB_2161029
AF405 anti-CD4 (RM4-5)	Thermo Fisher	Cat# MCD0426; AB_10373810
BV785 anti-CD45R/B220 (RA3-6B2)	BioLegend	Cat# 103246; AB_2563256
AF555 anti-IgA (polyclonal)	Southern Biotech	Cat# 1040-32; AB_2794378
AF594 anti-IgG (polyclonal)	Thermo Fisher	Cat# A-11005; AB_2534073
AF488 anti-MHCII (I-A/I-E) (M5/114.15.2)	BioLegend	Cat# 107616; AB_493523
APC/Cy7 anti-MHCII (I-A/I-E) (M5/114.15.2)	BioLegend	Cat# 107628; AB_2069377
**Chemicals, Peptides, and Recombinant Proteins**		
1-Thioglycerol	Sigma Aldrich	Cat# M1753
2-[4-(2-hydroxyethyl)piperazin-1-yl]ethanesulfonic acid (HEPES) (1 M)	Gibco	Cat# 15630056
2-Mercaptoethanol (50 mM)	Gibco	Cat# 31350010
Collagenase, from Clostridium histolyticum, Type I	Gibco	Cat# 17100017
D(+)-Surcrose	Sigma Aldrich	Cat# 84100
Dimethylsulfoxide (DMSO)	Sigma Aldrich	Cat# D2650
DNAse, from bovine pancreas, Type I	Roche	Cat# 11284932001
EDTA solution pH 8.0 (0.5 M) for molecular biology	PanReac AppliChem	Cat# A4892
Eosin B solution 1% in Ethanol	AppliChem	Cat# A9514
Ethyl cinnamate (ECi)	Sigma Aldrich	Cat# W243000
Fetal Calf Serum (FCS)	Gibco	Cat# 10270106
Fluoromount-G	Southern Biotech	Cat# 0100-01
Formalin Solution 10% (v/v), neutralised	Avantor	Cat# 3933.9020
G-DEX IIb RBC Lysis Buffer	iNtRON Biotechnology	Cat# 172R-1000
Hanks’ Balanced Salt Solution (HBSS), w/CaCl_2_, MgCl_2_	Gibco	Cat# 14025050
Hemalum solution acid acc. to Mayer	Carl Roth	Cat# T865.1
Histodenz	Sigma Aldrich	Cat# D2158
Hyaluronidase, from bovine testes, Type IV-S	Sigma Aldrich	Cat# H3884
Hydrogen Peroxide, 30% (v/v)	Carl Roth	Cat# CP26.5
IC Fixation Buffer	eBioscience	Cat# 00–8222–49
Isofluran CP	cp-pharma	Cat# 1214
Isopropanol	Carl Roth	Cat# CP41.2
N-Methylacetamide	Sigma Aldrich	Cat# M26305
Normal Mouse Serum	Jackson Immunoresearch	Cat# 015-000-120
PBS w/o Ca2+ Mg2+	Sigma Aldrich	Cat# D8537
Penicillin-Streptomycin (10,000 U/ml)	Gibco	Cat# 15140122
Roti-Histokitt II	Carl Roth	Cat# T160.1
RPMI 1640 Medium, GlutaMAX Supplement	Gibco	Cat# 61870010
Tissue-Tek O.C.T. Compound	Sakura Finetek	Cat# 4583
Triton X-100	Carl Roth	Cat# 6683.1
Xylene (isomeric mixture)	Merck	Cat# 1.08297
Zombie Aqua Fixable Viability Kit	BioLegend	Cat# 423101
**Software and Algorithms**		
FlowJo v10.2	BD Biosciences	SCR_008520
GraphPad Prism 7	GraphPad Software	SCR_002798
Fiji (ImageJ) 1.53f	https://fiji.sc/	SCR_002285
Imaris-to-FlowJo file conversion tool	This Manuscript	https://gitlab.com/kepplerlab/imaris_statistics_converter
Imaris v9.5	Bitplane	SCR_007370
Large Volume Computational Clearing (LVCC)	Leica Microsystems	
Leica Application Suite X	Leica Microsystems	SCR_013673
LIGHTNING	Leica Microsystems	www.leica-microsystems.com/products/confocal-microscopes/p/lightning/
**Other**		
µ-Slide 8 Well Glass Bottom	ibidi	Cat# 80827
Cover slips 24 × 60 mm # 1.5	Thermo Fisher	Cat# BB02400600AC13MNT0
Cover slips 8.5 × 8.5 mm # 1	Thermo Fisher	Cat# MENZBCAD00850085
EC ”Plan-Neofluar” 10×/0.30 M27	Zeiss	Cat#420340-9901
EC ”Plan-Neofluar” 20×/0.50 M27	Zeiss	Cat#420350-9900
FEATHER Disposable Scalpel No. 21	pfm medical	Cat# 200130021
HC PL APO CS2 20×/0.75 IMM objective	Leica Microsystems	Cat# 11506343
HC PL FLUOTAR 10×/0.32 DRY	Leica Microsystems	Cat# 11506522
HC PL FLUOTAR 63×/1.10 IMM	Leica Microsystems	
HC PL FLUOTAR L 20×/0.40 DRY	Leica Microsystems	
KIMTECH Science Precision Wipes	Kimberly-Clark Professional	Cat# 05511
N PLAN 5×/0.12 DRY	Leica Microsystems	
PARAFILM M Sealing Film	Carl Roth	Cat# H666.1
SuperFrost Plus Microscope Slides	Thermo Fisher	Cat# J1800AMNZ
Tissue-Tek Cryomold	Sakura Finetek	Cat# 4566
Type F Immersion liquid (RI = 1.518)	Leica Microsystems	Cat# 11513859

## Data Availability Statement

The original contributions presented in the study are included in the article/**Supplementary Material**. Further inquiries can be directed to the corresponding author.

## Ethics Statement

The animal study was reviewed and approved by Regierung von Oberbayern, Munich, Germany.

## Author Contributions

Conceptualization: SK. Methodology: SK. Investigation: JH, IG, RM, and SK. Formal Analysis: JH and SK. Writing—Original Draft: SK. Writing—Review and Editing: SK. Funding Acquisition: SK and JR. Resources: JR. Supervision: SK. All authors contributed to the article and approved the submitted version.

## Funding

This work was supported by the German Research Foundation (DFG) grant Ke1737/2-1 (SK), SFB 1054/B01—project number 360372040–SFB 1335/P01 and P08 (JR), SFB 1371/P05 (JR), TRR 237/A10 (JR), RU 695/9-1 (JR), the ERC (FP7, grant agreement No. 322865 awarded to JR) and the Else-Kröner-Fresenius-Stiftung grant 2019_A105 (SK).

## Conflict of Interest

The authors declare that the research was conducted in the absence of any commercial or financial relationships that could be construed as a potential conflict of interest.
