# Supplementary material for: Efficient Tissue Clearing and Multi-Organ Volumetric Imaging Enable Quantitative Visualization of Sparse Immune Cell Populations During Inflammation
Source: Front Immunol. 2021 Jan 25;11:599495. doi: 10.3389/fimmu.2020.599495 (PMC7869862; doi:10.3389/fimmu.2020.599495)
Supplement: Supplementary file 1 [file DataSheet_1.pdf]

**Inventory of Supplementary Material**

**Supplementary Figures:**

Supplementary Figure S1 - related to Figure 1

Supplementary Figure S2 - related to Figure 1

Supplementary Figure S3 - related to Figure 1

Supplementary Figure S4 - related to Figure 2

Supplementary Figure S5 - related to Figure 3

Supplementary Figure S6 - related to Figure 5

**Supplementary Videos:**

Video S1 - related to Figure 3

Video S2 - related to Figure 4

Video S3 - related to Figure 5

Video S4: - related to Figure 5

Video S5 - related to Figure 5

**Supplementary Legends:**

**Video S1 - related to Figure 3**

3D visualization of the EMOVI-treated kidney imaged by confocal microscopy also shown in Figure 3A and 3H. Field of view demonstrates distribution of MHC-II expressing cells surrounding or within glomeruli containing IgG deposits.

**Video S2 - related to Figure 4**

3D visualization of the EMOVI-treated kidney imaged by widefield microscopy using a 10x objective also shown in Figure 4B. CD31 staining depicted in grey demonstrates uniform labeling throughout the halved kidney.

**Video S3: - related to Figure 5**

3D visualization of the EMOVI-treated lymph node imaged by confocal microscopy also shown in Figure 5C. Image acquisition using a 20x objective allows detection of CD31 stained vessels throughout the lymph node (500 µm) in high resolution. Color coding indicates depth of vessel stain.

**Video S4: - related to Figure 5**

3D visualization of the EMOVI-treated lymph node imaged by widefield microscopy also shown in Figure 5D. Image acquisition using a 10x objective allows a rapid overview revealing distinct areas throughout the whole lymph node. CD21/35 staining in yellow depicts follicular dendritic cells marking B cell follicles, CD3 stain in cyan indicates T cell areas, CD31 in red depicts vasculature.

**Video S5 - related to Figure 5**

3D visualization of the EMOVI-treated piece of white adipose tissue (WAT) imaged by widefield microscopy also shown in Figure 5F. Image acquisition using a 20x objective allows detection of CD31 stained vessels (red) as well as MHC-II stained cells throughout the WAT piece. Autofluorescence in grey indicates outlines of adipocytes (dimensions: x = 1,8 mm. y = 1,9 mm, z (original 1 mm, cropped to z = 254,4 µm for display purposes).

## Supplementary Figure S1 - related to Figure 1

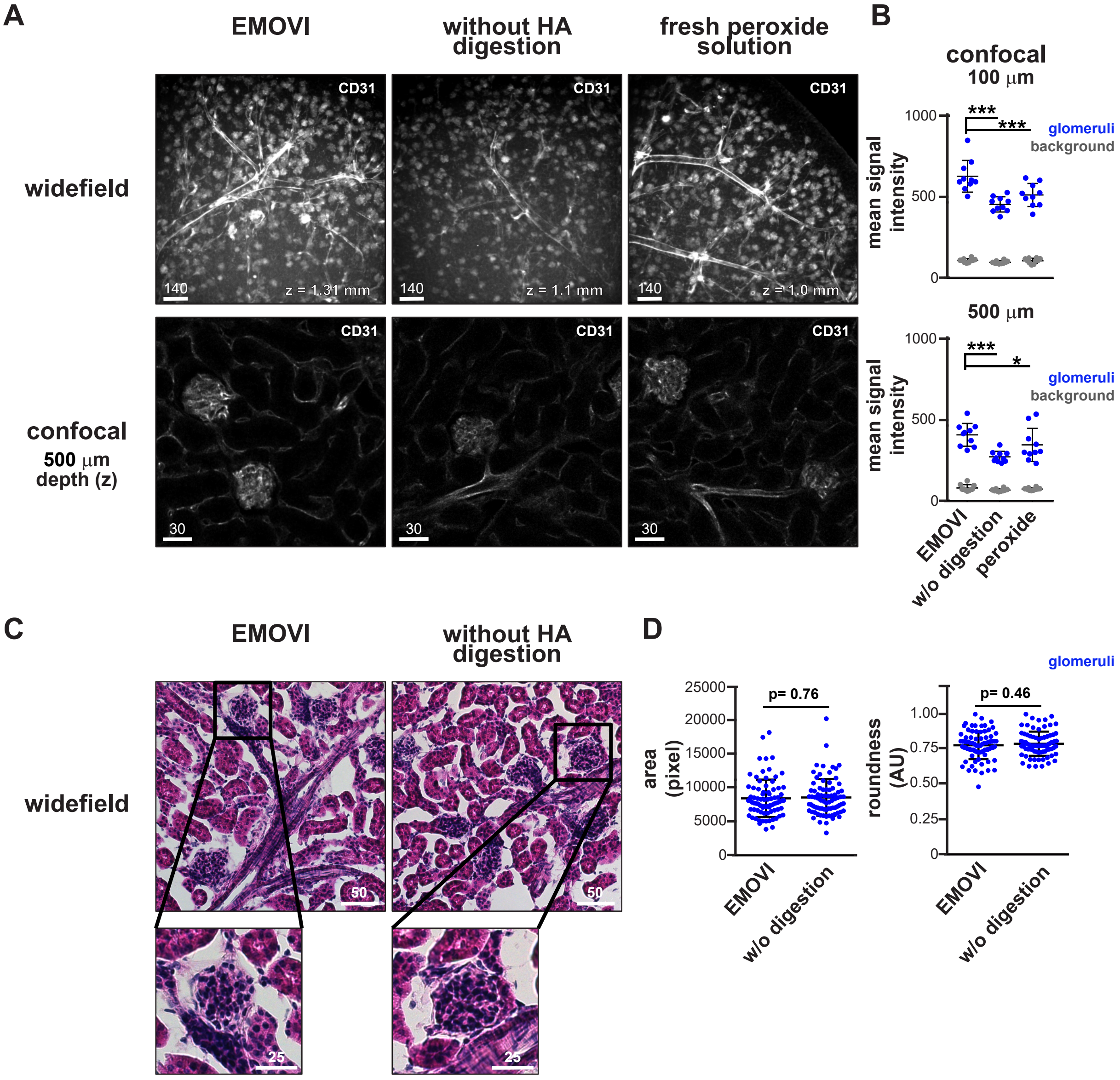


**Supplementary Figure S1 - related to Figure 1**
**Comparison of different staining and clearing approaches**
Perfused kidneys of the same mouse were fixed and halved to avoid variation between samples of different animals. All images were taken with the same settings using the widefield or confocal microscope respectively. All confocal images were not deconvolved. All widefield images were deconvolved enhancing differences of signal intensities to background between the methods. **(A+B)** Effect of hyaluronic acid (HA) digestion or freshly prepared peroxide solution on antibody penetrance and preservation of fluorescence. **(C)** Same kidneys as in **(A)** were de-cleared, rehydrated, OCT frozen, 10 µm sections cut and H&E stained. **(D)** Charts on the right depict area (pixels) and roundness of glomeruli (AU) in section overviews as determined by ImageJ. Each point depicts one glomerulus (n=80 each, mean +/- SD). Scale bars in µm. HA = hyaluronic acid. Representative images of 2 independent experiments are shown.

## Supplementary Figure S2 - related to Figure 1

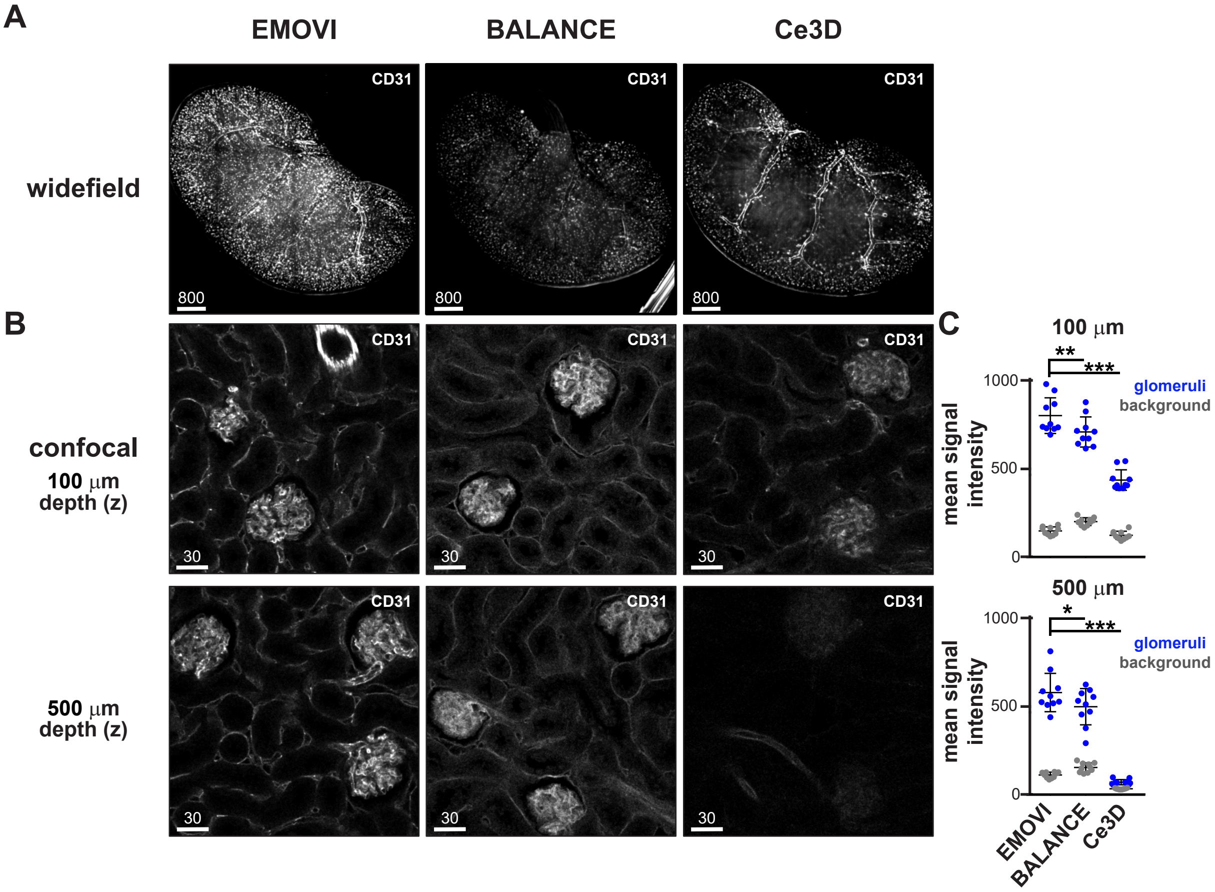


**Supplementary Figure S2 - related to Figure 1**
**Comparison of different staining and clearing approaches**
Perfused kidneys of the same mouse were fixed and halved to avoid variation between samples of different animals. All images were taken with the same settings using the widefield or confocal microscope respectively. All confocal images were not deconvolved. All widefield images were deconvolved enhancing differences of signal intensities to background between the methods. **(A)** Maximum intensity projections of halved kidneys with identical dimensions from one mouse stained and cleared with indicated methods and imaged using widefield microscopy. **(B)** Confocal images at indicated depth in Z (single plane) of the same kidneys as in **(A).**
Bar charts on the right **(C)** depict fluorescence intensities (mean grey values) in glomerular areas or non-glomerular areas (“background”) with similar size as determined by ImageJ on non-deconvolved confocal images. Each point depicts the fluorescence intensity per glomerulus or respective background (n=10 each, mean +/- SD). Scale bars in μm. Representative images of 2 independent experiments are shown.

## Supplementary Figure S3 - related to Figure 1

**A**

| organ | lung | lymph node | WAT | kidney | brain | liver | heart |
|---|---|---|---|---|---|---|---|
| **z in µm** | 82.2 | 187.2 | 68.6 | 139.4 | 188.2 | 84.3 | 132.1 |
| x | 581.25 | **x and y for all images the same as for lung** | | | | | |
| y | 581.25 | | | | | | |

**B**

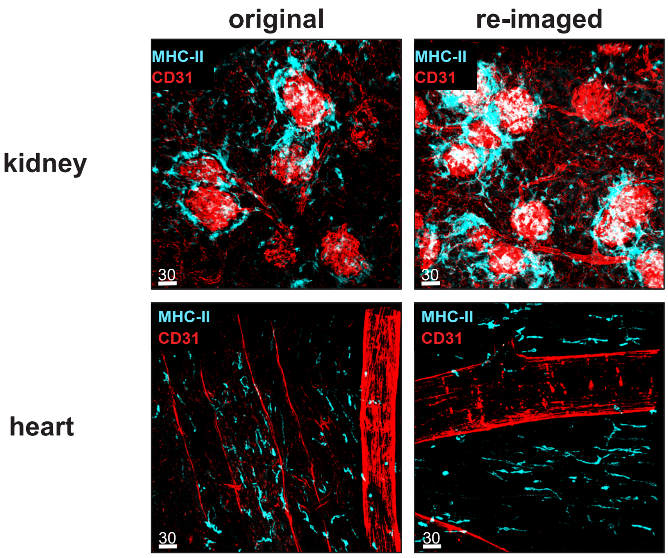


Kidney:

| laser line | original | re-imaged |
|---|---|---|
| 499 | 10 % | 10 % |
| 653 | 7.2 % | 7.2 % |
| x,y,z (µm) | 581.25, 581.25, 90.52 | 581.25, 581.25, 138.38 |

Heart:

| laser line | original | re-imaged |
|---|---|---|
| 499 | 8 % | 10 % |
| 653 | 20 % | 20 % |
| x,y,z (µm) | 581.25, 581.25, 131.1 | 581.25, 581.25, 69.55 |

**C**

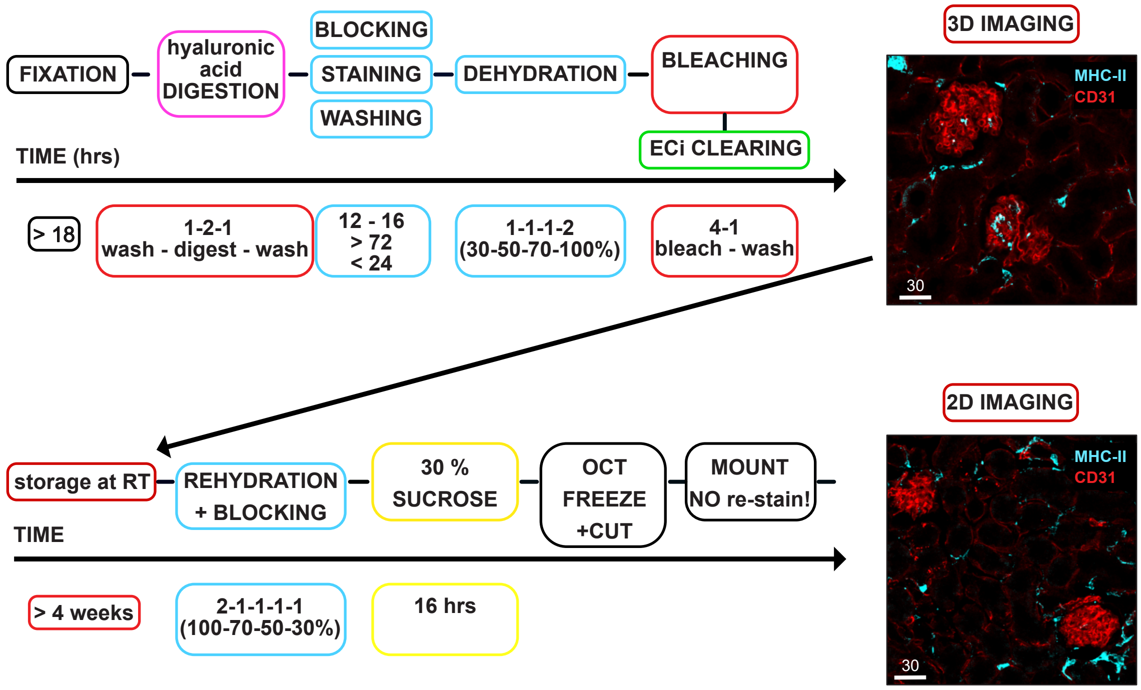


**Supplementary Figure S3 - related to Figure 1**
**(A)** Size (x,y,z) in µm of acquired images shown in Figure 1C. **(B)** Storage of tissue in ECi preserves antibody labeling over at least 4 month. Kidney and heart tissues were re-imaged after 4 months of storage at RT with similar laser settings as depicted in the tables shown on the right. **(C)** Organs can be de-cleared, re-hydrated and used for cryo-sectioning. Fluorescence of previously stained CD31 and MHC-II is still present. Scale bars in µm. Representative images of at least 3 independent experiments are shown.

## Supplementary Figure S4 - related to Figure 2

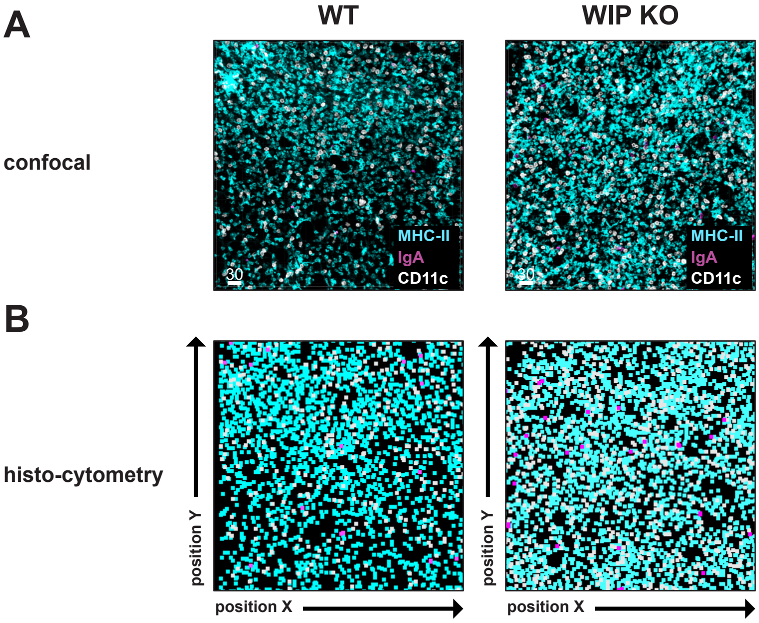


**Supplementary Figure S4 - related to Figure 2**
Lung tissue stained post-fixation with indicated antibodies allowed 3D visualization of single cells via confocal microscopy of a healthy or inflamed lung tissue **(A)** as well as **(B)** quantitative visualization of cellular positions. Scale bars in $\mu$m. Representative images of at least 2 independent experiments are shown.

## Supplementary Figure S5 - related to Figure 3

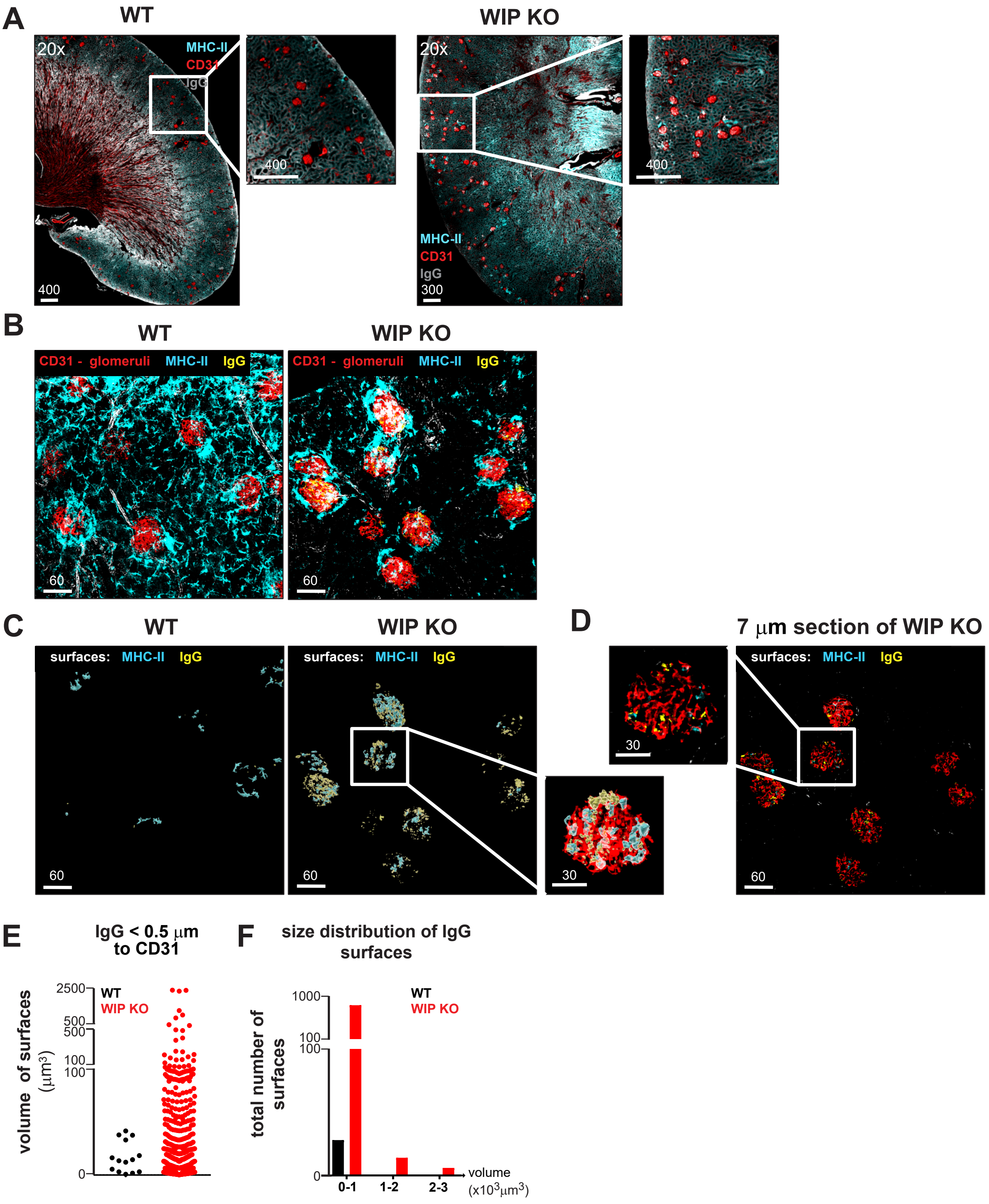


**Supplementary Figure S5 - related to Figure 3**
**3D imaging followed by histo-cytometry reveals cell infiltration of MHC-II positive cells in kidney glomeruli with IgG deposits**
**(A)** Kidney slices of a WT mouse or WIP KO mouse were stained with the indicated antibodies and an overview imaged by confocal microscopy using the LasX Navigator. **(B)** 3D surface creation by IMARIS was used to separately mask glomeruli and vessels from the CD31 stain. Staining of MHC-II expressing cells as well as IgG deposits is shown. **(C)** 3D surface creation by IMARIS was used to separately mask MHC-II expressing cells infiltrating glomeruli (distance <0.5 μm to glomeruli, cyan), and IgG deposits (distance <0.5 μm to glomeruli, yellow). **(D)** 7 μm thick sections traditionally used in histological sections reveal limited detection of IgG deposits and MHC-II expressing cells. **(E)** Surface volumes of IgG deposits with a distance of <0.5 μm to glomeruli as quantification for disease activity. **(F)** Size distribution of IgG surfaces. Scale bars in μm. Representative images of 2 independent experiments are shown.

## Supplementary Figure S6 - related to Figure 5

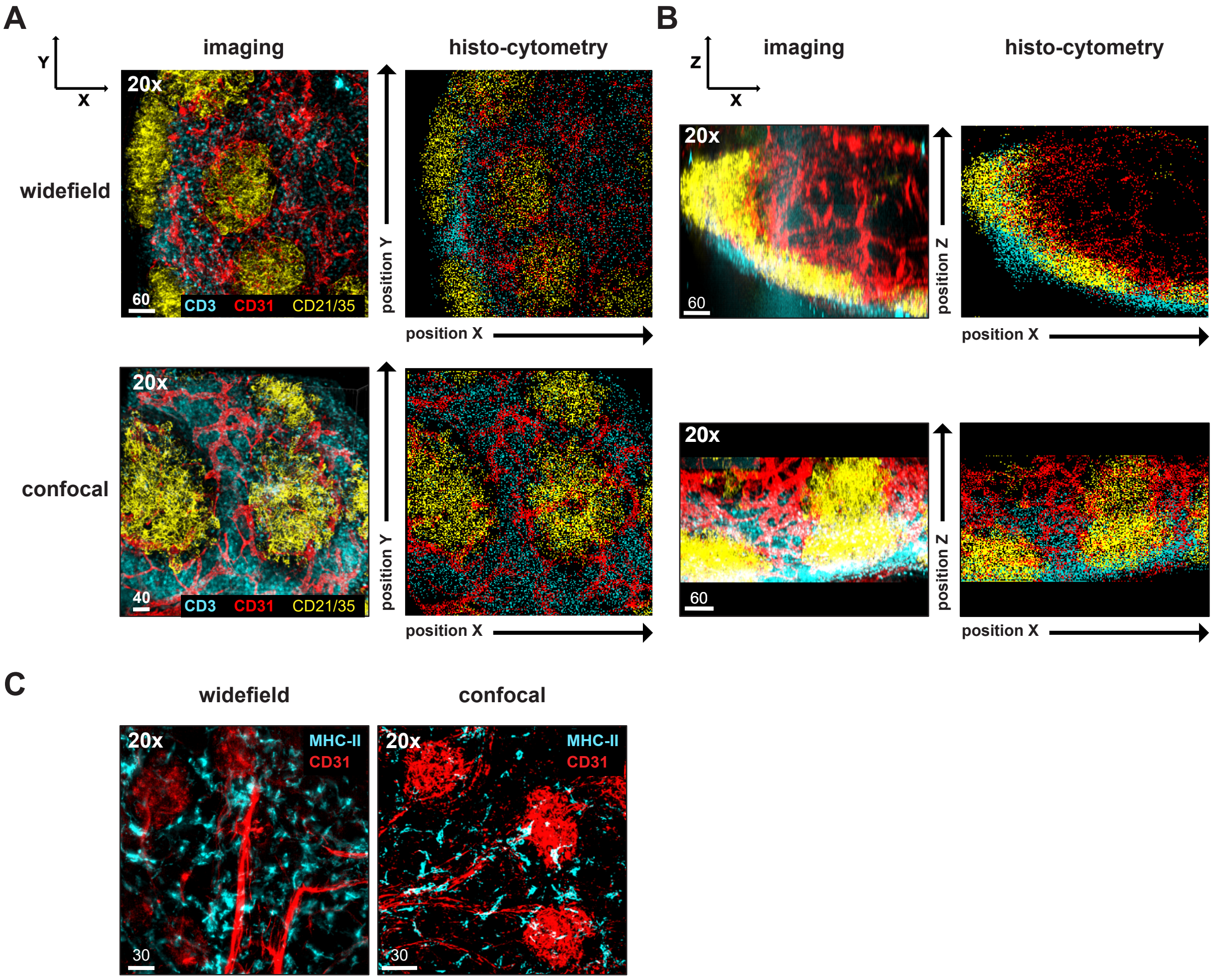


**Supplementary Figure S6 - related to Figure 5**
**(A)** Lymph nodes stained with the indicated antibodies, cleared with EMOVI and imaged by widefield (top) or confocal microscopy (bottom) with single cell resolution. Statistics for cellular objects were exported into FlowJo and used to visualize cellular positioning in FlowJo (histo-cytometry). **(B)** X and Z positions of same lymph nodes as in **A.** as imaged (left images) or cellular position as displayed using FlowJo (right images). **(C)** Widefield microscopy using a 20x objective reveals kidney glomeruli and MHC-II expressing cells. Confocal image acquired with a 20x objective (zoom factor of 1) on the right for comparison. Scale bars in µm. Representative images of at least 3 independent experiments are shown.
